# Nonsurgical Endodontic Retreatment of Advanced Inflammatory External Root Resorption Using Mineral Trioxide Aggregate Obturation

**DOI:** 10.1155/2012/624792

**Published:** 2012-12-11

**Authors:** Shivani Utneja, Gaurav Garg, Shipra Arora, Sangeeta Talwar

**Affiliations:** ^1^Department of Conservative Dentistry and Endodontics, Maulana Azad Institute of Dental Sciences, Bahadurshah Zafar Marg, New Delhi 110002, India; ^2^Department of Periodontics, Maulana Azad Institute of Dental Sciences, Bahadurshah Zafar Marg, New Delhi 110002, India

## Abstract

Inflammatory external root resorption is one of the major complications after traumatic dental injury. In this case report, we describe treatment of a maxillary central incisor affected by severe, perforating external root resorption. An 18-year-old patient presented with a previously traumatized, root-filled maxillary central incisor associated with pain and sinus tract. Radiographic examination revealed periradicular lesion involving pathologic resorption of the apical region of the root and lateral root surface both mesially and distally. After removal of the root canal filling, the tooth was disinfected with intracanal triple antibiotic paste for 2 weeks. The antibiotic dressing was then removed, and the entire root canal was filled with mineral trioxide aggregate. The endodontic access cavity was restored with composite resin. After 18 months, significant osseous healing of the periradicular region and lateral periodontium had occurred with arrest of external root resorption, and no clinical symptoms were apparent.

## 1. Introduction

Inflammatory external root resorption (IERR) is a pathologic condition caused by several etiologic factors including traumatic dental injury. Displacement of the teeth, as well as subsequent repositioning or replantation procedures inevitably causes damage to the root, resulting in denuded areas on the root surface which are chemotactic to phagocytes [1]. This inflammatory response can exacerbate in the presence of bacteria and their byproducts inside the root canal system and dentinal tubules after pulp necrosis and in the absence of protection of cementum barrier. If allowed to progress, the resorptive process may lead to rapid tooth loss [2, 3].

Clinically, teeth are usually not symptomatic in the early period of the process. However, as IERR progresses, the teeth may become symptomatic, and periradicular abscess may develop with increasing tooth mobility. Radiographically, radiolucencies can be observed in the external root surface and adjacent to the bone [4]. Endodontic treatment should be initiated promptly to prevent further hard tissue loss and root perforation [5]. In advanced cases, endodontic intervention may require prior repair of the perforation site with a suitable biomaterial [6].

Mineral trioxide aggregate (MTA) has emerged as a reliable bioactive material with extended applications in endodontics that include obturation of the root canal space owing to its superior physiochemical properties [7]. In conjunction with being sterile, radiopaque, and dimensionally stable the material is not sensitive to moisture and blood contamination [8]. MTA also provides an effective seal against dentin and cementum and promotes biologic repair and regeneration of the periodontal ligament [9, 10]. Clinical studies have confirmed the biocompatibility of this material and have shown a hard tissue inductive effect [11, 12]. These favorable properties render MTA a suitable material for the management of tissue damage caused by IERR [13]. Because perforation repair, root end induction, and root end filling are essential forms of partial canal obturation, the orthograde filling of the entire root canal system with MTA is the next logical progression in the evolutionary application of this material [14].

The present case report describes the nonsurgical endodontic retreatment of advanced IERR in a maxillary central incisor, which was treated with complete MTA obturation. 

## 2. Case Report

**Figure 1:**
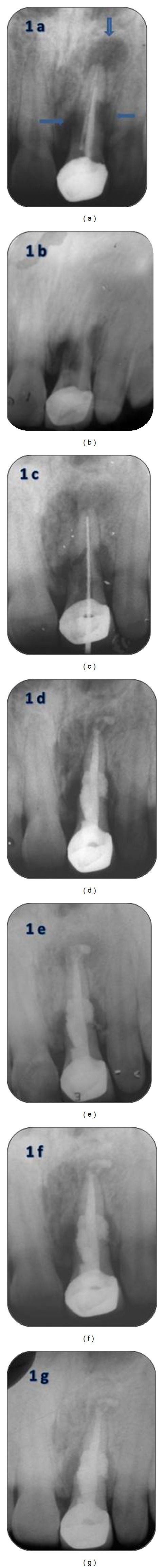
(a) Preoperative periapical radiograph showing periapical lesion and external apical and lateral root surface resorption in a maxillary left central incisor. The radiograph also reveals previous poor quality obturation. (b) Radiograph after removal of old gutta percha filling showing root canal outline completely visible through resorption defect. Bone resorption adjacent to resorption lacunae on root is also evident. (c) Working length radiograph. (d) Immediate postobturation radiograph showing adequate density of MTA obturation without voids and good adaptation of MTA to root contours. (e) Six-month followup periapical radiograph revealing stability of resorption and periapical healing. (f) Advanced osseous healing of periapical tissues and initiation of repair adjacent to lateral root surface resorption at 12 months. (g) An 18-month radiographic view showing complete regeneration of periradicular tissues and advanced osseous healing evident in the lateral periodontal tissues.

An 18-year-old male presented with the chief complaint of pain on mastication and recurrent swelling in the labio-gingival aspect of maxillary left central incisor. He gave a history of traumatic injury seven years earlier. For the first three years after trauma the patient did not take any treatment as the tooth remained asymptomatic. Thereafter the patient started experiencing pain in the tooth and underwent root canal treatment in 21 followed by full coverage restoration by a local dentist. Four years after root canal obturation the tooth again became symptomatic, and the patient was referred to our department for endodontic retreatment of 21. There was no relevant medical history. An intraoral examination revealed labial sinus tract with respect to 21. The tooth did not exhibit significant mobility, and there was no gingival sulcular bleeding on probing. 21 was tender to percussion. Radiographic examination of 21 showed extensive lateral and apical root resorption associated with periapical radiolucency and interdental bone loss both mesially and distally in relation to the resorption defect. The root canal space of 21 was inadequately obturated (Figure 1(a)).

Taking into consideration the extent and severity of the resorption, it was planned to do orthograde MTA obturation of the canal space after removing the old root canal filling to arrest the root resorption along the length of the root canal. This internal approach was preferred over surgical management as the latter would have sacrificed the already compromised bone support around 21. Intentional replantation was also not a good option as there was a danger of fracture of the tooth while extraction owing to severity of resorption. The procedure was fully explained to the patient, and informed consent was taken.

 The endodontic access opening was made through porcelain fused to metal crown using transmetal bur (Dentspy Maillefer, Ballaigues, Switerland). Previous gutta percha filling was removed using Hedstrom files in conjunction with Endosolv-E (Septodont, Inc., New castle, Delaware, USA). A radiograph taken to ensure complete removal of gutta percha showed uneven radiolucent regions along the external borders of the root, and the outline of original root canal was completely visible (Figure 1(b)). This was highly suggestive of external root resorption. Working length was determined radiographically (Figure 1(c)), and canal preparation was accomplished in a circumferential filing motion using H files while irrigating with copious amount of 1% sodium hypochlorite (Novo Dental Products Pvt. Ltd. Mumbai, India). This was followed by irrigation with normal saline to remove any remnants of hypochlorite. The canal was dried with absorbent paper points and filled with triple antibiotic paste (ciprofloxacin, metronidazole, and minocycline) as described by Sato et al. [15]. The access cavity was sealed with a temporary restorative material (Cavit-G, 3M ESPE, St. Paul, MN, USA). After 2 weeks, when the sinus had healed, intracanal medicament was flushed with distilled water and 1% sodium hypochlorite followed by rinse of saline. 2% chlorhexidine was used as the final irrigant. The canal was dried with absorbent paper points. White MTA (Pro Root Dentsply Tulsa, OK, USA) was mixed according to the manufacturer's instructions and was initially carried to the apical third using MTA carrier and compacted using endodontic plugger, size 5/7 (Dentsply, Maillefer, Ballaigues, Switzerland). After the apical 5 mm of canal was completely filled with MTA which was verified radiographically, a sterile reamer rolled with wet cotton was used to compact the MTA into the resorption cavity. In this way the entire root canal system was filled with MTA. A baseline radiograph was taken which showed that MTA was adequately compacted into the resorption defect with good adaptation to the root contours (Figure 1(d)). Moist cotton pellet was kept in the chamber, and the access cavity was sealed with a temporary restorative material. After one week, the temporary restoration was removed and the access cavity was restored with glass ionomer cement (Ketac Molar Easymix 3M ESPE).

The patient was recalled after 6, 12, and 18 months for clinical and radiographic followup. On clinical examination 21 was functional without sensitivity to percussion or palpation. The tooth showed normal physiologic mobility and no periodontal pockets on probing. The periapical radiograph showed regression in the size of periapical radiolucency with signs of osseous repair and no further progression of external resorption. There was initiation of osseous healing adjacent to mesial and distal resorption sites on the root surface (Figures 1(e), 1(f), and 1(g)). 

## 3. Discussion

IERR is initiated by mechanical trauma, resulting in the destruction of cementoblasts and loss of precementum and sometimes cementum in areas of the root surface. The resorptive process is then maintained by bacterial products from the infected root canal which provides the necessary continuous stimulation for the resorbing cells [1]. Thus, the treatment protocol for IERR should involve elimination of bacteria and their byproducts from the root canal system and dentinal tubules to stop the inflammatory processes involving the root surface to allow the regeneration of periodontium [16].

The conventional and preferred treatment protocol for a progressive IERR consists of chemomechanical preparation of the root canal system including a short-term (1 month) dressing of creamy paste of calcium hydroxide (Ca(OH)_2_) for bacterial disinfection of the root canal space. The process is followed by a long-term dressing of densely packed Ca(OH)_2_ to provide an alkaline Ph inside the dentinal tubules to kill the bacteria and neutralize the endotoxins, which are potent inflammatory stimulators [4, 17].

Although this treatment protocol has a high success rate [1], the long-term use of Ca(OH)_2_ has some disadvantages. Because the treatment includes repeated clinical sessions to replace Ca(OH)_2_, it demands high cooperation and motivation from the patient. In addition, long-term presence of Ca(OH)_2_ in root canal space can increase the brittleness of the root dentin and the risk of future cervical root fractures especially in immature teeth [18]. There are studies that discuss the contraindication of Ca(OH)_2_ use in teeth with damaged cementum because of the necrotizing effects of Ca(OH)_2_ on periodontal cells that repopulate on the root surface. This necrotizing effect can prevent formation of a normal attachment apparatus and result in replacement resorption and ankylosis [19].

The antibacterial and tissue dissolution action of sodium hypochlorite increases with its concentration, but this is accompanied by an increase in toxicity [20]. As the contact between the irrigant and the surrounding vital tissues cannot be completely avoided in cases of perforating resorption, therefore a dilute concentration of the irrigant that still retains adequate disinfective properties is recommended [21]. In the present case 1% w/v sodium hypochlorite did not cause any caustic effects on periodontal tissues. To further prevent its prolonged contact with surrounding vital tissues, normal saline was used each time after rinsing the canal with sodium hypochlorite [22]. Final rinse before obturation was performed using 2% chlorhexidine solution as the present case was a retreatment case where high proportions of gram-positive bacteria are suspected. Hence use of chlorhexidine as a final irrigant would appear advantageous in such a situation [20]. Placement of an intracanal medicament is crucial in necrotic cases. In our case a mixture of ciprofloxacin, metronidazole, and minocycline was chosen as an intracanal medicament as it has been shown to be very effective in eliminating endodontic pathogens in vitro and in situ [15]. Apart from disinfection, minocycline present in this paste might have also contributed in arresting the inflammatory resorption as it possesses anti-inflammatory property. This has been proved in reimplanted dog teeth after extended dry time that intracanal placement of ledermix paste which contains corticosteroid and tetracycline inhibits inflammatory root resorption [23]. 

The development of new bioactive materials such as MTA make possible other therapeutic approaches, including the obturation of the root canal space in complex cases of iatrogenic or pathologic root perforation [14]. In the present case, there was severe inflammatory external resorption in the lateral and apical portions of the root associated with periapical pathology. As the tooth was very fragile, treating it by orthograde obturation of the entire canal system with MTA was the only viable option. It has been shown that intracanal application of MTA can also cause release of calcium ions through dentinal tubules into external resorption defect, which may favour the repair potential of the surrounding tissues [24]. Recent research has demonstrated that root canal treated teeth obturated with MTA exhibit higher fracture resistance than their untreated counterparts [14]. This could be attributed to the ability of MTA to prevent the destruction of collagen by inducing the expression of a tissue inhibitor of metalloproteinase 2 in the dentin matrix [25].

In the case described here 6, 12, and 18 month radiographic recall showed osseous repair of periapical pathosis. Although reestablishment of the entire periodontal apparatus did not occur, the resorption site remained stable throughout the 18-month followup period. The patient was asymptomatic, with the tooth exhibiting normal sulcular probing depth, mobility, and function. The patient was also pleased with the treatment outcome as a permanent tooth with otherwise hopeless prognosis was maintained. Orthograde retreatment of the present case with MTA proved to be very conservative as it lead to avoidance of surgical treatment with similar prognostic outcomes. However, longer followup for this case is required to be sure of success.
